# Annexin A1 Released in Extracellular Vesicles by Pancreatic Cancer Cells Activates Components of the Tumor Microenvironment, through Interaction with the Formyl-Peptide Receptors

**DOI:** 10.3390/cells9122719

**Published:** 2020-12-18

**Authors:** Nunzia Novizio, Raffaella Belvedere, Emanuela Pessolano, Alessandra Tosco, Amalia Porta, Mauro Perretti, Pietro Campiglia, Amelia Filippelli, Antonello Petrella

**Affiliations:** 1 Department of Pharmacy, University of Salerno, Via Giovanni Paolo II 132, 84084 Fisciano, Italy; nnovizio@unisa.it (N.N.); rbelvedere@unisa.it (R.B.); epessolano@unisa.it (E.P.); tosco@unisa.it (A.T.); aporta@unisa.it (A.P.); pcampiglia@unisa.it (P.C.); 2 The William Harvey Research Institute, Barts and The London School of Medicine and Dentistry, Queen Mary University of London, London EC1M 6BQ, UK; m.perretti@qmul.ac.uk; 3 Department of Medicine, Surgery and Dentistry, University of Salerno, Via S. Allende 43, 84081 Baronissi, Italy; afilippelli@unisa.it

**Keywords:** extracellular vesicles, exosomes, annexin A1, pancreatic cancer, FPRs

## Abstract

Pancreatic cancer (PC) is one of the most aggressive cancers in the world. Several extracellular factors are involved in its development and metastasis to distant organs. In PC, the protein Annexin A1 (ANXA1) appears to be overexpressed and may be identified as an oncogenic factor, also because it is a component in tumor-deriving extracellular vesicles (EVs). Indeed, these microvesicles are known to nourish the tumor microenvironment. Once we evaluated the autocrine role of ANXA1-containing EVs on PC MIA PaCa-2 cells and their pro-angiogenic action, we investigated the ANXA1 paracrine effect on stromal cells like fibroblasts and endothelial ones. Concerning the analysis of fibroblasts, cell migration/invasion, cytoskeleton remodeling, and the different expression of specific protein markers, all features of the cell switching into myofibroblasts, were assessed after administration of wild type more than ANXA1 Knock-Out EVs. Interestingly, we demonstrated a mechanism by which the ANXA1-EVs complex can stimulate the activation of formyl peptide receptors (FPRs), triggering mesenchymal switches and cell motility on both fibroblasts and endothelial cells. Therefore, we highlighted the importance of ANXA1/EVs-FPR axes in PC progression as a vehicle of intercommunication tumor cells-stroma, suggesting a specific potential prognostic/diagnostic role of ANXA1, whether in soluble form or even if EVs are captured in PC.

## 1. Introduction

Pancreatic cancer (PC) remains one of the deadliest cancers worldwide, mainly due to the limited response to currently available forms of treatments, as demonstrated by the frequently reported poor clinical outcome and high mortality rate [1,2]. In previous studies, comparative analysis of protein profiles of PC and normal pancreatic cells have already highlighted a significant over-expression of Annexin A1 (ANXA1) [3,4]. This protein retains calcium-mediated phospholipid binding properties and participates in many physiopathological processes including cell proliferation, migration, differentiation and death as well as inflammation [5,6,7]. In tumor development, ANXA1 can be considered as a tissue-specific oncogene or oncosuppressor [8,9,10]. During PC progression, it may contribute to the acquisition of a mesenchymal phenotype by tumor cells, essential for their diffusion in distant sites, suggesting that the protein may represent a potential PC diagnostic/prognostic marker [11,12]. 

The different functions attributed to ANXA1 are related to its subcellular localization, for example, inside the cells, ANXA1 can directly and indirectly promote the cytoskeletal organization [13,14,15]. ANXA1 can also be secreted in the extracellular environment, even though the mechanisms by which this occurs are still not well-defined [13]. Furthermore, it has been found that in different cancer cells including PC cells, ANXA1 is released in both full-length and truncated forms, after having undergone post-transductional modifications [14,16]. Studies investigating the mechanisms of ANXA1 secretion have found that this protein is also involved in the biogenesis of extracellular vesicles (EVs). As a component of these microvesicles enriched in exosomes (40–100 nm diameter), the protein further contributes to the aggressiveness of PC [17]. Nevertheless, despite the specific mechanism of interaction of ANXA1/EVs with target cells remaining poorly understood [18], it has been elucidated that the autocrine loop created by ANXA1 on origin/recipient cells is triggered by the protein interaction with the formyl-peptide receptors (FPRs). The latter are well known receptor partners for ANXA1 on immune and tumor cells [19,20]. Moreover, the activation of the axis ANXA1-FPRs leads to the migratory responses of PC cells [11,14,15,17,18]. 

In this study we focused on the paracrine effects of EVs from PC cells, having already shown the significant contribution of ANXA1 to the in vitro angiogenesis [17]. Indeed, the EVs, mainly exosomes, seem to play a relevant role in cell-to-cell communication in several physiopathological processes, including the formation of pre-metastatic niches, thus favoring tumor progression [21,22]. During the phases of tumor development EVs carry out a pivotal role in creating the contact between PC cells and stroma [23,24,25]. Therefore, we hypothesized that the ANXA1-EVs complex could stimulate the activation of FPRs on fibroblasts and endothelial cells [26,27,28,29], triggering mesenchymal switches and cell motility. Thus, the importance of the axis ANXA1/EVs-FPRs in PC progression was assessed from a different point of view, and specifically considering the interplays between tumor cells and stroma. So, in this work the tumor microenvironment has been represented by human fibroblasts and endothelial cells. Hence, based on the knowledge that these two populations are interconnected with other ones, such as mesenchymal stem cells and immune cells, future efforts will focus on the evaluation of a more complex in vivo system, by which we could clarify the potential role of ANXA1 as a prognostic/diagnostic soluble factor, even EVs captured, in the PC model.

## 2. Material and Methods 

### 2.1. Cell Culture 

MIA PaCa-2 cells (ATCC CRL-1420; Manassas, VA, USA) were cultured in Dulbecco’s Modified Eagle Medium (DMEM) containing L-glutamine 2 mM, 10% heat-inactivated fetal bovine serum (FBS), 10,000 U/mL penicillin, and 10 mg/mL streptomycin (Euroclone; Milan, Italy). ANXA1 knock-out (KO) MIA PaCa-2 cells were created from the wild type (WT) cells through the CRISPR/Cas9 genome editing system, as reported in [11], and kept in selection by 700 μg/mL neomycin (Euroclone; Milan, Italy). BJ cell line (human immortalized fibroblasts, ATCC CRL2522TM) were cultured in Eagle’s Minimum Essential Medium (MEM) with 10% FBS, 1% L-glutamine, 1% sodium pyruvate, 1% NEAA and antibiotics. HUVEC cell line (Human umbilical vein endothelial cell) (ATCC^®^ PCS-100-010^TM^, Manassas, VA, USA) was maintained as reported in [12,30] until passage 10. All cells were maintained at 37 °C in a 5% CO_2_-95% air humidified atmosphere. 

### 2.2. Exosome Enrichment 

The enrichment of exosomes (to which we will generally refer to as extracellular vesicles, EVs) obtained from cell culture supernatants was performed as reported in [31]. Briefly, the WT and ANXA1 KO MIA PaCa-2 cells (1.5 × 10^5^ cm^−2^, for a total of about 8 × 10^7^ cells), after abundant washing with phosphate buffered saline (PBS), were incubated for 24 h in DMEM medium without FBS. The conditioned medium was thus collected and centrifuged the first time for 5 min at 300× *g* at room temperature (RT) to remove the detached cells. It was recovered and centrifuged again for 10 min at 2000× *g* at 4 °C to remove dead cells, after which it was centrifuged once more at 10,000× *g* for another 30 min at 4 °C to remove cellular debris. The supernatant was transferred in tubes and ultracentrifugated for 70 min at 100,000× *g* at 4 °C. Subsequently, the pellet was washed in PBS and re-ultracentrifuged at 100,000× *g* at 4 °C for 70 min. Finally, the supernatant was removed and the pellet was re-suspended according to the experimental use.

The amount of exosomes administered to the cells was normalized at 20 μg of WT and ANXA1 KO MIA PaCa-2 EVs through the Bradford assay, as reported in [17]. The normalization allowed for the administration of the same amount of EV to the cells, derived from the WT and ANXA1 KO MIA PaCa-2 cells, in all phases of the experiment. All analyses were performed on fresh isolated fractions.

### 2.3. Western Blotting 

Proteins extracted from cells and EVs were examined by Sodium Dodecyl Sulphate - PolyAcrylamide Gel Electrophoresis (SDS-PAGE). Protein content was estimated according to the Biorad protein assay (BIO-RAD, Hercules, CA, USA), as previously described [17]. We have analyzed primary antibodies against rabbit polyclonal ANXA1 (1:10,000; Invitrogen; Carlsbad, CA, USA), calreticulin (1:1000; Elabscience; Houston, TX, USA), and mouse monoclonal TSG101 (1:1000; ThermoFisher Scientific; Waltham, MA, USA), CD81 (1:200; Becton Dickinson Labware, Franklin Lakes, NJ, USA), CD63 (1:200; Biolegend; San Diego, CA, USA), and GAPDH (mouse monoclonal, 1:1000; Santa Cruz Biotechnologies, Dallas, TX, USA). The blots were exposed to Las4000 (GE Healthcare Life Sciences; Little Chalfont, UK).

### 2.4. Wound-Healing Assay

A wound was produced on the confluent monolayer of BJ and HUVEC by scraping the cells with a pipette tip. Subsequently, the cells were treated according to the experimental points. Mitomycin C (10 μg/mL, Sigma-Aldrich; Saint Louis, MO, USA) was further added to ensure the block of mitosis. The wounded cells were analyzed as reported in [32]. The values we show represent the average of the measured distances of five different positions for which ten cells were selected on both sides of the wound. 

### 2.5. Invasion Assay

BJ and HUVEC invasiveness was studied using the trans-well cell culture (12 mm diameter, 8.0-fim pore size) purchased form Corning Incorporated (New York, NJ, USA), as previously described [30,33]. In the lower chamber of each well were added the treatments as established in the experimental points. Mitomycin C (10 μg/mL, Sigma-Aldrich; Saint Louis, MO, USA) was included to ensure the arrest of mitosis. Staining and analysis procedures were performed as reported in [34].

### 2.6. Confocal Microscopy 

BJ and HUVEC cells, fixed in p-formaldehyde (4% *v*/*v* in PBS; Lonza; Basilea, Swiss), were permeabilized with Triton X-100 (0.5% *v*/*v* in PBS; Lonza; Basilea, Swiss), blocked with goat serum (20% *v*/*v* in PBS; Lonza; Basilea, Swiss). Next, cells were incubated O/N at 4 °C with antibodies against VEGF (rabbit polyclonal, 1:100; Santa Cruz Biotechnologies, Dallas, TX, USA), αSMA (rabbit polyclonal, 1:100; Cusabio Life Science, College Park, MD, USA), VE-cadherin (mouse monoclonal, 1:100; Santa Cruz Biotechnologies, Dallas, TX, USA), FAP1α (rabbit polyclonal, 1:100; Santa Cruz Biotechnologies, Dallas, TX, USA), fibronectin (mouse monoclonal, 1:100; Abcam, Cambridge, UK), Col1A (mouse monoclonal, 1:100; Santa Cruz Biotechnologies, Dallas, TX, USA), FGF-2 (rabbit polyclonal, 1:100; Santa Cruz Biotechnologies, Dallas, TX, USA), vimentin (mouse monoclonal, 1:250; Santa Cruz Biotechnologies, Dallas, TX, USA), and vinculin (mouse monoclonal, 1:100; Santa Cruz Biotechnologies, Dallas, TX, USA). F-actin was evaluated by 5 μg/mL of Phalloidin-FITC (Sigma-Aldrich; Saint Louis, MO, USA) for 30 min, at RT in the dark. The staining with anti-mouse and anti-rabbit antibodies and the nuclei and the confocal analysis were performed as described in [17]. 

### 2.7. Gelatin Gel Zymography 

SDS-PAGE zymography allowed us to detect gelatinolytic activity as reported in [35]. Briefly, the samples, serum-free supernatants, were prepared in a standard non-reducing loading buffer for SDS-PAGE. The 0.1% gelatin substrate (for protease detection) was incorporated into the resolution gel during the preparation of the 10% polyacrylamide gel. After electrophoresis, performed at 125 V, the SDS was removed from the gel by incubation in renaturing solution (2.5% Triton X-100) for 1 h, followed by incubation in a special digestion buffer (50 mM of Tris-HCl, pH 7.8, 200 mM of NaCl, 5 mM of CaCl_2_, and 5 mM of ZnCl_2_) for an optimized period of time, at 37 °C, which allows the degradation of the substrate. The gel was subsequently colored with Coomassie Brilliant Blue R-250 and then bleached with destaining solution (10% methanol and 5% acetic acid). The areas of digestion appeared as light bands on a dark stained background where the substrate was degraded by the enzyme.

### 2.8. Tube Formation Assay 

As reported in [36], Matrigel (Becton Dickinson Labware, Franklin Lakes, NJ, USA) mixed with EGM-2 1:1 was seeded in a 24-well plate on ice and incubated at 37 °C for 30 min to allow for gelation. HUVEC cells were added to the top of the gel at a density of 2 × 10^4^ cells/well in the presence or absence of the active principles as indicated for each experimental point. The cells were incubated at 37 °C with 5% CO_2_. After 12 h, the images were captured using the EVOS^®^ optical microscope (10×) (Life technologies Corporation, Carlsbad, CA, USA). Subsequent analyzes, both for the length of each tube and the number of branches, were carried out using ImageJ software (NIH, Bethesda, MD, USA) (angiogenesis analyzer for ImageJ).

### 2.9. Flow Cytometry 

BJ and HUVEC cells were harvested at a number of 1 × 10^6^ and analyzed for FPR-1 and FPR-2, as reported in [36]. Briefly, pellets were incubated for 1 h at RT in PBS 1x containing APC-conjugated anti-human antibody against FPR-1 (1:250, R&D system, Minneapolis, MN, USA) or PE-conjugated anti-human antibody against FPR-2 (1:250, R&D system, Minneapolis, MN, USA). The cells were analyzed with a Becton Dickinson FACScan flow cytometer (Franklin Lakes, NJ, USA) using the Cells Quest program. 

### 2.10. RNA Isolation and Quantitative Real Time- Polymerase Chain Reaction (RT-PCR)

mRNA levels of BJ and HUVEC cells were analyzed by real-time PCR using the Light Cycler 480 II instrument (Roche, Indianapolis, IN, USA). One µg of total RNA extracted from cells with Trizol reagent was reverse transcribed into cDNA with Transcriptor First Strand cDNA Synthesis Kit (Roche, Indianapolis, IN, USA). cDNAs were processed using Light Cycler 480 Syber Green I Master mix and primers for *FPR*-1 (Bio-Fab research; Rome, Italy) (5′-GGTGAACAGTCCAGGAGCAG-3′, 3′-ACCTCCCTGTGGACGACATA-5′), *FPR*-2 (Bio-Fab research; Rome, Italy) (5′-CTGGCTACACTGTTCTGCGG-3′, 3′-AGCACCACCTGTAGTTGGAG-5′), and Hypoxanthine Phosphoribosyltransferase 1 (*HPRT*1) (Bio-Fab research; Rome, Italy) (5′-GACCAGTCAACAGGGGACAT-3′, 3′-CCTGACCAAGGAAAGCAAAG-5′) following the manufacturer’s protocols. Results were analyzed using the Delta-Delta CT method. A portion (10 µL) of the RT-PCR product was electrophoresed on a 2% agarose gel in a Tris-acetate-EDTA buffer. The gel was stained with ethidium bromide and exposed to Las4000 (GE Healthcare Life Sciences; Little Chalfont, UK).

### 2.11. Statistical Analysis

Data analysis and statistical evaluations were made with Microsoft Excel. For each experiment, the number of independent repetitions and p values in the legends of the figures were indicated. All results are the mean ± standard deviation of at least three different experiments performed in technical triplicate. The statistical data between the experimental points were obtained thanks to the one-way ANOVA tool. The differences were considered significant if *p* < 0.05, *p* < 0.01 and *p* < 0.001.

## 3. Results

### 3.1. Effects of Extracellular Vesicles (EVs) from Wild type (WT) and ANXA1 Knock-Out (KO) MIA PaCa-2 Cells on Fibroblast Migration and Invasion

**Figure 1:**
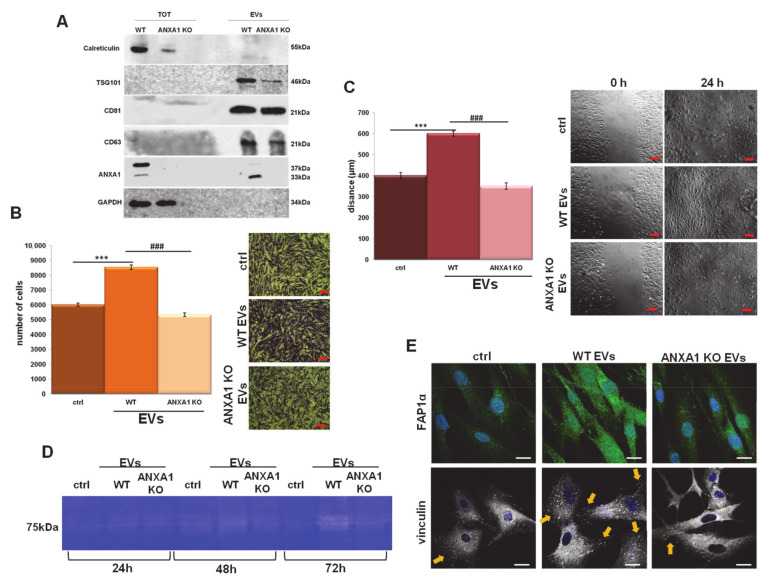
MIA PaCa-2 extracellular vesicles (EV) effects on BJ cells. (**A**) Western blot using antibodies against calreticulin, TSG 101. CD63, CD81, and ANXA1 on the protein content of total cell lysates and EV fractions extracted from the WT and ANXA1 KO MIA PaCa-2 cells. Protein normalization and the check of the sample quality were performed on GAPDH levels. Analysis of (**B**) invasion and (**C**) migration distance of BJ cells treated with EVs from wild type (WT) and ANXA1 knock-out (KO) MIA PaCa-2 cells with relative bright field images. Bar = 50 μm. (**D**) Gelatin zymography showing increased gelatinolytic activity of MMP-2 of BJ cells. Zymography was performed using 0.1% gelatin gel as described in Section 2, followed by Coomassie blue staining. (**E**) Immunofluorescence analysis to detect Fibroblast Activated Protein 1α (FAP1α) and vinculin. Nuclei were stained with Hoechst 33342 1:1000 for 30 min at room temperature (RT) in the dark. Magnification 63 × 1.4 numerical aperture (NA). Bar = 100 μm. Data represent the mean of three independent experiments ± standard deviation with similar results. *** *p* < 0.001 treated cells vs. untreated controls; ### *p* < 0.001 for each point of ANXA1 KO MIA PaCa-2 cells vs. WT one.

In order to investigate the role of ANXA1 on EVs-dependent metastatic potential of PC cells, we studied the paracrine effects of EVs derived by WT and ANXA1 KO MIA PaCa-2 cells on BJ fibroblasts, one of the cellular components of the tumor microenvironment [37,38]. First, the EVs obtained from PC MIA PaCa-2 cells were characterized by the presence of TSG101 or the absence of calreticulin, used as positive and negative controls respectively, as previously described [17]. We also showed the exclusive EVs and abundant expression of tetraspanins CD63 and CD81, often used for the detection of secreted microvesicles [39]. Our results also confirmed the different expression of ANXA1, as revealed by the lack of ANXA1 KO total and EV protein extracts, for both full length and cleaved forms (Figure 1A).

Once validated, the EV effects on HUVEC motility [17], the BJ cell migration was analyzed by wound-healing assay as well as the capability of invasion through the coating of Matrigel in transwells. As shown in Figure 1B,C, BJ cells invaded and migrated more rapidly in the presence of EVs isolated from WT MIA PaCa-2 cells when compared to those obtained from ANXA1 KO cells and to the untreated controls. In order to support the results obtained with the invasion assay, we performed a gel zymography to assess the activity of metalloproteinases 2 (MMP2) (e.g., gelatinolytic enzymes), secreted by the cells. The degrading activity of BJ MMP2 was significantly increased in the presence of EVs from WT MIA PaCa-2 compared to the EVs released by ANXA1 KO cells. This kind of signal appeared evident after 48 and 72 h of treatment (Figure 1D).

Furthermore, the activation of fibroblasts triggers their switch into myofibroblasts. This phenomenon is related to the increase in the expression of specific protein markers, particularly relevant of which is the fibroblast activated protein 1α (FAP1α), a member of the group II integral serine proteases, and vinculin, a cytoskeletal protein of the focal adhesion plaques [40,41,42]. BJ cells showed a notable increase of levels of FAP1α expression when treated with EVs from WT MIA PaCa-2, more than ANXA1 KO EV. The WT EV treatment triggered a marked increase of traction stresses, with an increase in the expression of vinculin not only with respect to the control cells, but also with respect to the ANXA1 KO EV treated cells. Furthermore, by this confocal analysis, we proved that fibroblasts acquired more precise and parallel directionality in the presence of WT EVs than in the presence of ANXA1 KO EV treated cells (Figure 1E).

### 3.2. Ac2-26 Peptide Promoted Fibroblasts and Endothelial Cells Motility through Formyl Peptide Receptors (FPRs)

It has been observed that the secreted form of ANXA1 stimulates a more aggressive phenotype in cancer cells by its interaction with FPRs [14,15,16].

**Figure 2:**
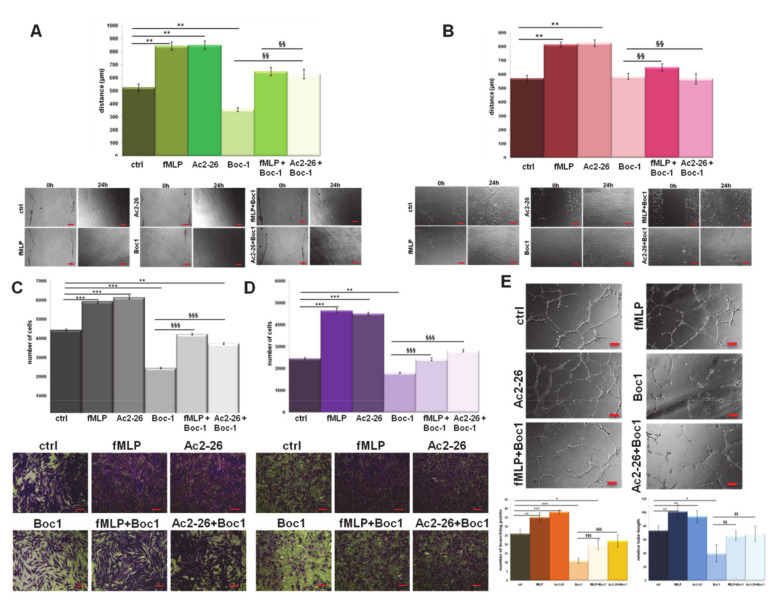
Effects of Formyl peptide receptors (FPRs) agonists and antagonists on BJ and Human umbilical vein endothelial cell (HUVEC) cells. Results of Wound-Healing assay on (**A**) BJ and (**B**) HUVEC cells treated with formyl-Methionine-Leucine-Phenylalanine (fMLP) (50 nM), Ac2-26 (1 µM), and N-tert-butyloxycarbonyl-Met-Leu-Phe (Boc1) (100 µM), with related images. Analysis of invasion distance of BJ (**C**) and HUVEC (**D**) with relative bright field images treated or not with FPR agonists and antagonist at the same concentration. Bar = 50 μm. (**E**) Representative images of tube formation by HUVEC cells seeded for 12 h on Matrigel: Endothelial Cell Growth Basal Medium-2 (EBM-2) 1:1 with the same treatment. Analysis of tube length and number of branches calculated by ImageJ (Angiogenesis Analyzer tool) software. Bar = 100 μm. Data represent the mean of three independent experiments ± standard deviation with similar results.* *p* < 0.05; ** *p* < 0.01; *** *p* < 0.001 treated cells vs. untreated controls; §§ *p* < 0.01; §§§ *p* < 0.001 for each point of EVs + Boc-1 vs. Boc-1 alone.

Particularly, the N-terminal mimetic peptide of ANXA1, Ac2-26, is known to be involved in cell migration and invasion by acting on these receptors [5,6,11,14]. In this study, we assessed these processes on BJ and HUVEC cells, showing an increase in migration distance (Figure 2A,B, for BJ and HUVEC cells, respectively) and invasion ability (Figure 2C,D, for BJ and HUVEC cells, respectively) of cells treated with Ac2-26 or with the natural FPR agonist fMLP (formyl-Methionine-Leucine-Phenylalanine), compared to the controls. The FPR antagonist Boc-1 (*N*-*tert*-butyloxycarbonyl-Met-Leu-Phe), at a concentration greater than 1 micromolar, acts as a pan-antagonist on the known FPR isoforms [43], significantly inhibiting the basal and Ac2-26 or fMLP-stimulated migration.

Another aspect considered was the in vitro angiogenesis on HUVEC cells. These cells showed a higher tendency to form capillary-like structures when treated with Ac2-26 and fMLP to a greater extent than the control cells. Additionally, Boc-1 has the ability to influence angiogenic activity negatively, with or without treatment (Figure 2E). These events have been further corroborated by the analysis of the number of branching points and the relative tube length calculated as reported in Section 2. 

### 3.3. PC Cells-EVs Interact with FPRs on Human Fibroblasts

**Figure 3:**
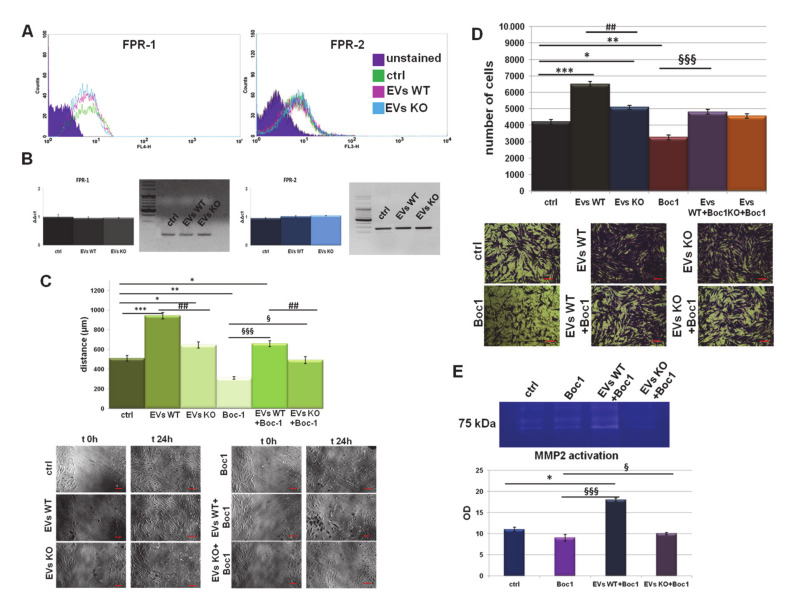
EVs interact with FPRs on fibroblasts. (**A**) Expression of FPR-1 and FPR-2 on BJ cells were analyzed by flow cytometry. The violet areas in the plots are relative to unstained fibroblasts. FPR-1 and FPR-2 signals are showed in green for ctrl, in pink for WT EVs and in light blue for ANXA1 KO EVs treated cells for 24 h. (**B**) Real Time- Polymerase Chain Reaction (RT-PCR) for *FPR*-1 and *FPR*-2, mRNA expression measured on levels of *HPRT*1. Values refer to the same experimental points of flow cytometry analysis and are expressed using the delta-delta Ct method to derive relative fold change. It is also shown the electrophoresis of the *FPR*-1 and *FPR*-2 RT-PCR products on 2% agarose gel stained with ethidium bromide. Results of Wound-Healing assay and (**C**) invasion analysis with relative bright field images of BJ cells, treated or not with EVs from WT and ANXA1 KO MIA PaCa-2 cells and Boc1 (100 µM). Bar = 50 μm. (**D**) Gelatin zymography showing gelatinolytic activity of MMP-2 of BJ supernatants. (**E**) Data represent the mean of four independent experiments ± standard deviation with similar results.* *p* < 0.05; ** *p* < 0.01; *** *p* < 0.001 treated cells vs. untreated controls; ## *p* < 0.01; for each point of ANXA1 KO MIA PaCa-2 cells vs. WT one; § *p* < 0.05; §§§ *p* < 0.001 for each point of EVs + Boc-1 vs. Boc-1 alone.

Once evaluated, the activation of FPRs, the mechanism by which PC cells-deriving EVs might induce the motility of human fibroblasts, was investigated. First, we assessed FPR-1 and FPR-2 expression, referred to as the better characterized receptor isoforms [43], on BJ cells by cytofluorimetric analysis. The receptor expression did not change in the presence of WT and ANXA1 KO EVs (Figure 3A). We confirmed these data through the RT-PCR performed using the cDNA obtained from fibroblasts treated with WT EVs and ANXA1 KO EVs for 24 h compared to the not treated control (Figure 3B).

Next, we examined the migration and invasion distances of these cells treated with EVs derived from WT and ANXA1 KO MIA PaCa-2 cells with or without Boc-1. The results in Figure 3C,D show that fibroblasts treated with WT EVs, together with Boc-1, migrated and invaded more than the control, but less than the WT EV treated cells. The motility effects were inhibited by co-administration of ANXA1 KO EVs plus Boc-1. Actually, the rescue was not significant as for the WT EVs, above all for the invasion assay (Figure 3C,D). Finally, to support the results obtained by the invasion assay, we performed gel zymography reporting the activation of MMP2 in BJ cell supernatants. Gel degradation increased in the presence of WT EVs and Boc-1 if compared to the not treated and Boc-1 treated cells, but also when the FPR-antagonist was co-administered ANXA1 KO EVs (Figure 3E). 

### 3.4. ANXA1-Containing EVs Interact with FPRs on Endothelial Cells

**Figure 4:**
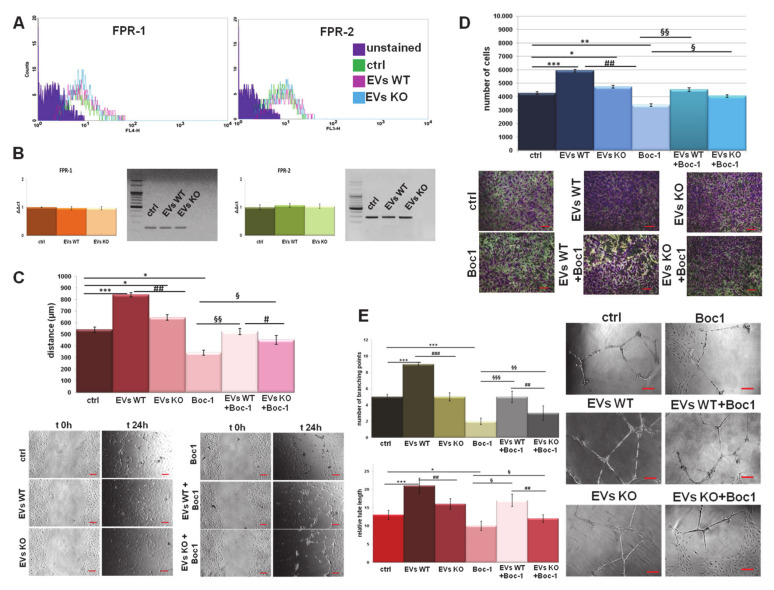
EVs interact with FPRs on endothelial cells. (**A**) Expression of FPR-1 and FPR-2 on HUVEC cells was analyzed by flow cytometry. The violet areas in the plots are relative to unstained fibroblasts. FPR-1 and FPR-2 signals are showed in green for ctrl, in pink for WT EVs and in light blue for ANXA1 KO EVs treated cells for 24 h. (**B**) RT-PCR for *FPR*-1 and *FPR*-2, mRNA expression measured on levels of *HPRT*1. Values refer to the same experimental points of flow cytometry analysis are expressed using the delta-delta Ct method to derive relative fold change. It is also shown the electrophoresis of the *FPR*-1 and *FPR*-2 RT-PCR products on 2% agarose gel stained with ethidium bromide. Results of HUVEC (**C**) migration and (**D**) invasion in presence of WT and ANXA1 KO EVs with or without Boc-1 (100µM) with relative bright field images. Bar = 50 μm. (**E**) Representative images of tube formation by HUVEC cells with the related analysis of tube length and number of branches. Bar = 100 μm. Data represent the mean of four independent experiments ± standard deviation with similar results. * *p* < 0.05; ** *p* < 0.01; *** *p* < 0.001 for treated cells vs. untreated controls; # *p* < 0.05, ## *p* < 0.01; ### *p* < 0.001 for each point of ANXA1 KO MIA PaCa-2 cells vs. WT one; § *p* < 0.05; §§ *p* < 0.01; §§§ *p* < 0.001 for each point of EVs + Boc-1 vs. Boc-1 alone.

The paracrine effects of PC cell-derived EVs on endothelial cells has already been investigated [17]. Here, we focused on the possible mechanism of these phenomena. As for human fibroblasts, FPR-1 and FPR-2 expression also remained unmodified in HUVEC cells in the presence of both WT and ANXA1 KO EVs (Figure 4A). In this case, this result was also confirmed by RT-PCR (Figure 4B). Additionally, as shown for the BJ cells, EVs also induced a signal transduction via FPRs in HUVEC cells. The addition of Boc-1 to WT EVs determined a significant slowdown of the migration and invasion distances compared to cells treated with WT EVs alone. The same trend, but much less pronounced, was observed when Boc-1 was added ANXA1 KO EVs, similar to BJ cells (Figure 4C,D). Finally, the inhibition of FPRs through Boc-1 also presented a significant effect on in vitro angiogenesis. Indeed, it has been found that the rescue of WT EV action is more relevant than ANXA1 KO EVs (Figure 4E), as highlighted by the bright field images and the calculation of the number of branching points and of the relative tube length.

### 3.5. WT EVs Promoted the Fibroblast Switch More than ANXA1 KO EVs

**Figure 5:**
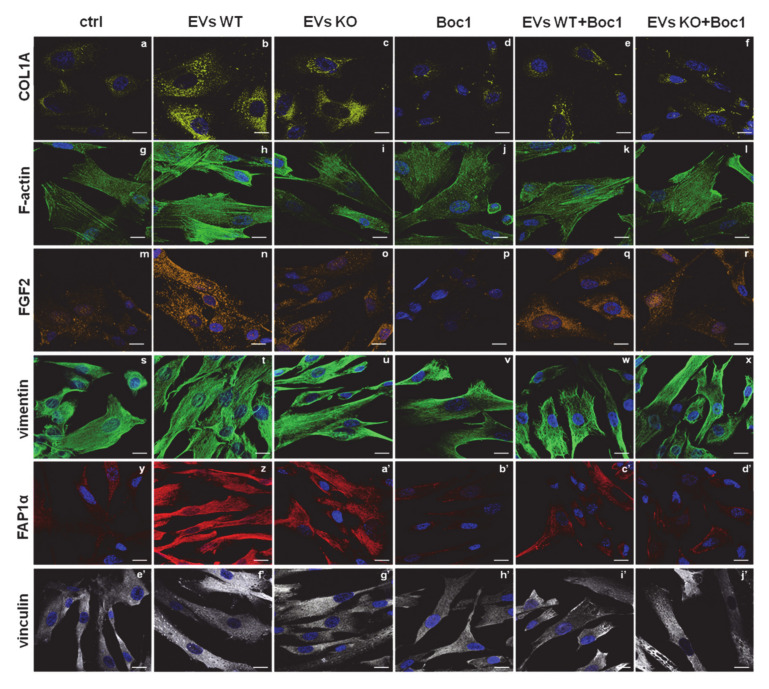
Fibroblasts activation induced by ANXA1-containing EVs. Immunofluorescence analysis on BJ cells to detect: COL1A (panels **a**–**f**), F-actin (panels **g**–**l**), FGF2 (panels **m**–**r**), vimentin (panels **s**–**x**), FAP1α (panels **y**–**d’**) and vinculin (panels **e’**–**j’**). Nuclei were stained with Hoechst 33,342 1:1000 for 30 min at RT in the dark. Magnification 63 × 1.4 NA. Bar = 100 µm.

The tumor microenvironment could have relevant effects on PC aggressiveness and on the development of a malignant phenotype of cancer cells [44,45]. In this scenario, fibroblasts can differentiate into myofibroblasts, forming a tumor-responsive stroma, under the effects of soluble factors and exosomes produced by tumor cells [46]. In order to investigate this switch, we analyzed some of the principal mesenchymal markers. In Figure 5, BJ cells showed a notable increase of expression levels of FAP1α, collagen type I alpha1 (COL1A) and fibroblasts growth factor 2 (FGF2) when treated with EVs from WT MIA PaCa-2 than when treated with ANXA1 KO EVs (panels y-a’, a-c and m-o, respectively). In the presence of Boc-1, the levels of these proteins appeared strongly reduced (panels b’, d, p, respectively). When this antagonist has been administered together with ANXA1 KO EVs, the signal for these markers became very similar to the untreated cells (panels d’, e and q, respectively), while an intermediate intensity was revealed in the case of Boc-1/WT EVs (panels c’, d, and p).

Based on the increased migration and invasion rates, our analyses of BJ cells further focused on the cytoskeletal organization. Here, we found a significant increase of well-organized stress fibers, as revealed by phalloidin staining of F-actin, when the cells were treated with WT EVs. Actin polymerization reached an intermediate situation between ctrl and WT EVs when BJ was treated with ANXA1 KO EVs. Again, Boc-1 negatively affected this process and interfered more with WT EVs than with ANXA1 KO EVs (panels g–l). A very similar trend was observed for vinculin expression, for which the WT and ANXA1 KO EVs confirmed its increase (panels e’–g’). On the other hand, Boc-1 determined a net inversion of the formation of vinculin adhesion plaques. Moreover, in the case of co-administration, Boc-1 plus WT EVs, this inversion appeared higher than the addition of the mix Boc-1/ANXA1 KO EVs (panels h’–j’). Furthermore, the expression of vimentin, one of the intermediate filaments, a crucial mesenchymal marker, resulted in not being affected by the treatments. Interestingly, this protein appeared much better structured in the presence of WT EVs than in the presence of ANXA1 KO EVs. Vimentin organization was strongly affected by Boc-1 added together with WT EVs than with ANXA1 KO EVs (panels s–x). Finally, the confocal analysis allowed us to show an elongated form and a parallel-organized pattern in almost 100% of the seeded population in the presence of WT EVs, but less with ANXA1 KO EVs. The untreated cells and the cells treated with Boc-1 resulted in being disorganized and randomly orientated. As far as the expression markers are concerned, the co-administration of EVs and Boc-1 led to an intermediated behavior.

### 3.6. WT EVs Are Able to Induce the EndMT through FPRs

In direct response to vesicles released from a primary tumor, the endothelium can undergo the endothelial-to-mesenchimal transition (EndMT), acquiring a mesenchymal phenotype [47,48]. EndMT may play an important role in stabilizing the neovasculature during angiogenesis. For this reason, we used confocal microscopy to investigate the effects of EVs from WT and ANXA1 KO MIA PaCa-2 cells on morphological features and on the expression of some proteins involved in this process in HUVEC cells.

**Figure 6:**
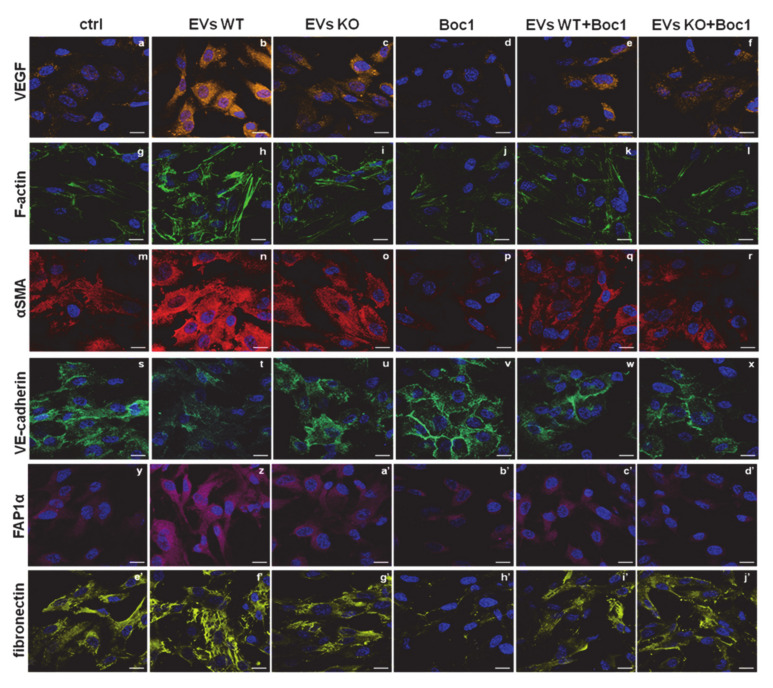
EndMT induced by ANXA1-containing EVs. Confocal analysis for HUVEC cells in the presence of WT and ANXA1 KO EVs with and without Boc-1 for: VEGF (panels **a**–**f**), F-actin (panels **g**–**l**), αSMA (panels m–r), VE-cadherin (panels **s**–**x**), FAP1α (panels **y**–**d’**), fibronectin (panels **e’**–**j’**). Nuclei were stained with Hoechst 33,342 1:1000 for 30 min at RT in the dark. Magnification 63 × 1.4 NA. Bar = 100 μm.

In Figure 6, in the presence of WT EVs, HUVEC disclosed a greater expression of vascular endothelial growth factor (VEGF), α-smooth muscle actin (αSMA), FAP1α, and fibronectin (panels a–b, m–n, y–z, and e’–f’, respectively), considered altogether as markers of EndMT [32,36]. Furthermore, the expression of these proteins underwent a smaller increase in the presence of EVs from ANXA1 KO clones (panels c, o, a’, g’, respectively). Accordingly, Boc-1 was more effective when FPRs were stimulated by WT EVs than by ANXA1 KO (panels d–f, p–r, b’–d’, h’–j’, respectively, for VEGF, αSMA, FAP1α, fibronectin). Again, vascular endothelial (VE)-cadherin showed an increased cytosolic expression with WT EVs than with ANXA1 KO EVs; Boc-1 blocked these effects and the protein remained linked to cell membrane despite the vesicles’ stimulation (panels s–x). Finally, the phalloidin staining allowed us to show the F-actin fibers more sharply with WT EVs compared to ANXA1 KO EVs. The intermediate behavior for each related treatment was reached in the presence of the FPR antagonist, as for the other markers considered (panels g–l).

## 4. Discussion

The PC microenvironment plays a critical role in tumor progression [49], revealing a strong relationship between its organization and metastasis. The tissue surrounding the PC cells consists of the same cancer cells and stromal cells (e.g., fibroblasts, endothelial cells, immune cells, and extracellular components such as EVs that sustain the primary tumor in an autocrine and paracrine manner. EVs, particularly if enriched in exosomes, are functional carriers necessary to guarantee cell–cell communication and their content can have strong effects on the recipient cells. Interesting evidence has demonstrated that in many models of cancer including PC [50], exosomes contribute to tumor progression by the formation of pre-metastatic niches [51,52]. This finding has raised much interest and led many researchers to implement studies aiming to increase the knowledge of these structures, like the analysis of proteomic content performed by Yu et al. Among the most significant elements, ANXA1 was identified as one of the proteins associated with PC metastasis in multiple organs, mainly in the liver [53]. In parallel, our previous works demonstrated that ANXA1, in its intracellular and extracellular forms, promotes PC progression by respectively acting as a cytoskeletal remodeling factor and as an agonist of FPRs [11,14]. Moreover, in our last work, we showed that ANXA1 is a key actor of EVs isolated from MIA PaCa-2 PC cells, revealing its importance in the production and/or secretion of these microvesicles [17].

In the present paper, based on the fact that (i) the extracellular microvesicles have an important role in PC progression, (ii) ANXA1 is involved in the exosomes biogenesis and/or effects, and (iii) the extracellular counterpart of this protein is able to trigger cell motility [5,6,14,54,55], we decided to investigate the in vitro mechanism of action of ANXA1/EVs on fibroblasts and endothelial cells as recipient stromal cells. The use of the ANXA1 KO MIA PaCa-2, compared to WT one, was confirmed to be a good in vitro model to preliminarily describe the action of the protein of our interest on tumor microenvironment.

The different degree of fibroblast activation depending on the presence ANXA1 in PC deriving EVs was analyzed, showing their switch into myofibroblasts-like features, as highlighted by the induced cell motility, metalloproteinases action, and increased expression of FAP1α and vinculin.

The interaction of the secreted form of ANXA1 with a FPR receptor partner family is known, as commonly observed in both physiological and pathological models [22,56,57,58,59]. This binding is able to trigger calcium mobilization, actin polymerization, adhesion, invasion, and focused migration [5,6,11,14,54]. Therefore, we used molecules such as fMLP and Ac2-26 as endogenous ligands and Boc-1 as an antagonist to show the main biological events triggered by FPRs, leading to a more aggressive cell phenotype. Once the expression and activation of the receptor isoforms 1 (FPR1) and 2 (FPR2) on both BJ and HUVEC cells was tested, it was possible to show the mechanism by which the EVs obtained from MIA PaCa-2 were able to bring about a significant variation in the recipient cell behavior, especially if containing ANXA1. These activities were assessed in terms of the acquisition of a mesenchymal phenotype, which is instrumental for tumor metastasis [11,60]. Fibroblasts are known to play an important role in cancer progression, in terms of chemoresistance, a consistent physical block against chemotherapeutic agents, and because they can switch into cancer associated fibroblasts (CAFs), mimicking the features of myofibroblasts [61,62,63,64,65]. This phenotype is generally represented by a well-organized rearrangement of the cytoskeleton in a parallel cell orientation. In this study, the action of ANXA1 in EVs on FPRs was assessed by the inhibitory effect on the migratory and invasive behavior in the presence of the receptors pan-inhibitor Boc-1. Hence, this result demonstrates that Boc-1 is able to block the ANXA1 effects, like the classical FPRs agonists, even if this protein is part of the microvesicles. Moreover, these particular responses leading to a mesenchymal phenotype have also been examined by evaluating the increased expression of COL1A, FGF2, vinculin, and FAP1α. The confirmation of this switch has also been derived from the assessment of a more structured orientation of vimentin and F-actin. Particularly, the negative effect of Boc-1 appeared more evident when it was added together with WT EVs more than ANXA1 KO EVs, further underlining an important involvement of ANXA1 on fibroblast behavior. On the other hand, the involvement of both ANXA1 and tumor-derived EVs as pro-angiogenic elements, already widely described in literature [18,66,67,68,69,70], confirm the previously described interaction of EV-containing ANXA1 with FPRs [17]. In particular, here we report the main biological changes triggering angiogenesis such as cell functional migration and invasion and EndMT [18], a process evaluated by the increase in several protein markers such as VEGF, αSMA, FAP1α, and fibronectin and the loss of plasma membrane localization of the adhesion molecule VE-cadherin. All these aspects have been modified in the presence of Boc-1 more extensively in the case of WT MIA PaCa-2 cells-deriving EVs compared to ANXA1 KO cells, highlighting once more the participation of ANXA1 in EVs on FPRs and also on endothelial cells.

In this study, another important issue brought about was the involvement of EVs as a vehicle of ANXA1 externalization, which for a long time has been considered as essential for the disclosure of protein behavior [13]. On the other hand, up to today, the precise mechanism of interaction of ANXA1/EVs with target cells remains controversial. In this scenario, as suggested for other biological systems [56], we propose that the presence of ANXA1 on the external side of the vesicle membrane allows these to directly interact with FPRs, triggering the activation of receptor-related downstream events. However, it is still not clear whether the EVs are endocytosed by the receiving cells as an alternative or even an additional mechanism. Thus, this issue can certainly represent a point for future studies focused on the identification of ANXA1 as a potential target for therapy/prevention of PC dissemination. Furthermore, we could suggest that this FPR-dependent cascade is able to induce a positive loop, culminating in the increase of microvesicles production and release, which could favor tumor development.

Generally, the extracellular form of ANXA1 has been detected in human sera in several conditions. In the case of inflammation, for example, ANXA1 containing-EVs have been proven to raise pro-repair and anti-inflammatory effects and to represent a diagnostic biomarker. Moreover, the prospect of ANXA1-containing nanoparticles to deliver therapeutic benefit has also been investigated [71]. Additionally, the identification of ANXA1 as a circulating molecule in the sera of cancer-affected patients has been considered as prognostic factor because of its correlation with clinicopathological conditions [72]. Thus, this protein maintains all the features of an appealing target for PC therapy.

Finally, further investigations are necessary to confirm the role of the ANXA1-EV complex deriving from PC in the metastatic process and engraftment in distant organs. Particularly, we propose extending the analysis to other cell populations surrounding the primary tumor such as macrophages and immune cells. Another point of interest will be furnished by in vivo investigation, exploiting not only the influence of EVs deriving from WT and/or ANXA1 KO MIA PaCa-2 engrafts, but also the effects of direct implanting of these microvesicles in animal models.

## Abbreviations

PC	Pancreatic cancer
ANXA1	Annexin 1
EVs	Extracellular vesicles
FPRs	Formyl peptide receptors
ATCC	American Type Culture Collection
DMEM	Dulbecco’s Modified Eagle Medium
FBS	Fetal bovine serum
KO	Knock-out
WT	Wild type
MEM	Eagle’s Minimum Essential Medium
NEAA	Non-Essential Amino Acid
HUVEC	Human umbilical vein endothelial cell
PBS	Phosphate buffered saline
RT	Room temperature
SDS-PAGE	Sodium Dodecyl Sulphate - PolyAcrylamide Gel Electrophoresis
RT-PCR	Real Time- Polymerase Chain Reaction
HPRT1	Hypoxanthine Phosphoribosyltransferase 1
TSG101	Tumor susceptibility gene 101
GAPDH	Glyceraldehyde-3-Phosphate Dehydrogenase
VEGF	Vascular Endothelial Growth Factor
αSMA	α-Smooth Muscle Actin
FAP1α	Fibroblast Activated Protein 1α
Col1A	Collagen type I alpha1
FGF-2	Fibroblasts Growth Factor 2
FITC	Fluorescein isothiocyanate
EGM-2	Endothelial cell growth medium
MMP2	Metalloproteinases 2
fMLP	formyl-Methionine-Leucine-Phenylalanine
Boc1	N-tert-butyloxycarbonyl-Met-Leu-Phe
EndMT	Endothelial-to-Mesenchimal Transition
